# 中国年轻肺腺癌患者基因特点研究

**DOI:** 10.3779/j.issn.1009-3419.2020.101.17

**Published:** 2020-04-20

**Authors:** 宇 梁, 和磊 侯, 曼 姜, 传涛 张, 栋 刘, 晓春 张

**Affiliations:** 266003 青岛，青岛大学附属医院肿瘤科 Department of Medical Oncology, The Affiliated Hospital of Qingdao University, Qingdao 266003, China

**Keywords:** 肺腺癌, 年轻, 高通量测序, 基因图谱, Lung adenocarcinoma, Young age, Next-generation sequencing, Genetic profile

## Abstract

**背景与目的:**

全球肺癌的发病率正呈逐年上升趋势，其中腺癌所占的百分比日益升高。据统计，全球的肺癌平均初诊年龄在70岁左右，虽然肺癌仍以老年患者居多，但发病年龄的年轻化趋势愈加明显。结合现有研究数据，我们已知在非小细胞肺癌中，年轻患者疾病的发生有其独特的生物学特点。但年轻肺腺癌患者的基因组学特性和临床特征仍有待确定。本研究采用高通量测序（next-generation sequencing technology, NGS）技术对中国年轻肺腺癌患者的基因突变状态进行了研究。

**方法:**

共收集了89例年龄≤45岁的肺腺癌患者组织标本，所有患者均知情同意。使用NGS检测用于确定癌组织中驱动基因突变。此外，对同期行NGS检测的95例 > 45岁肺腺癌患者的基因组和临床病理特征进行回顾性分析。

**结果:**

根据年龄分类对184例肺腺癌患者的驱动基因突变频率进行了分析，揭示了年龄≤45岁的年轻组患者的独特基因特征。其中间变淋巴瘤激酶（anaplastic lymphoma kinase, *ALK*）融合基因和人表皮生长因子受体-2（human epidermal growth factor receptor 2, *HER2*）基因的突变频率较高。而鼠类肉瘤病毒癌基因（kirsten rat sarcoma viral oncogene, *KRAS*）、丝氨酸/苏氨酸蛋白激酶11（serine/threonine kinase 11, *STK11*）和表皮生长因子受体（epidermal growth factor receptor, *EGFR*）20外显子突变的趋势则相反，这些突变在年龄 > 45岁的老年组中更为常见。此外，年轻组*EGFR*基因突变同时伴有肿瘤蛋白p53（tumor protein p53, *TP53*）基因突变较老年组更为普遍（81.6% *vs* 44.9%），这可能使其应用EGFR酪氨酸激酶抑制剂（EGFR-tyrosine kinase inhibitor, EGFR-TKI）后疗效较差。

**结论:**

NGS分析显示年轻腺癌患者具有独特的基因突变特点。在年轻患者中发现*EGFR/TP53*共突变的频率较高，这些独特的基因组学特征对临床治疗有重要的指导意义。

## Abstract (en)

**Background and objective:**

Occurrence at a younger age has been demonstrated to be associated with a distinct biology in non-small cell lung cancer. However, genomics and clinical characteristics among younger patients with lung adenocarcinoma remain to be determined. Here we studied the potentially targetable genetic alterations by next-generation sequencing (NGS) assay in young Chinese patients with lung adenocarcinoma.

**Methods:**

Eighty-nine surgically resected lung adenocarcinoma tissue samples from patients aged less than 45 years were collected with informed consent from all patients. Targeted NGS assays were used to identify actionable genetic alterations in the cancer tissues. Additionally, the genomic and clinical pathologic characteristics of 95 patients with lung adenocarcinoma who received NGS testing over the same period were analyzed retrospectively.

**Results:**

The frequencies of targetable genetic alterations in 184 patients with lung adenocarcinoma were analyzed by defined age categories, which unveiled a distinctive molecular profile in the younger group, aged less than 45 years. Notably, higher frequency of anaplastic lymphoma kinase (*ALK*) and human epidermal growth factor receptor 2 (*HER2*) genetic alterations were associated with young age. However, a reverse trend was observed for kirsten rat sarcoma viral oncogene (*KRAS*), serine/threonine kinase 11 (*STK11*) and epidermal growth factor receptor (*EGFR*) exon 20 mutations, which were more frequently identified in the older group, aged more than 45 years. Furthermore, concurrent *EGFR*/tumor protein p53 (*TP53*) mutations were much more prevalent in the younger patients (81.6% *vs* 44.9%), which might have a poor response to treatment with EGFR-tyrosine kinase inhibitor (EGFR-TKI).

**Conclusion:**

NGS assay revealed a distinctive genetic profile in younger patients with adenocarcinoma. High frequency of concurrent *EGFR/TP53* mutations was found in the younger patients, which especially warranted personalized treatment in this population.

非小细胞肺癌（non-small cell lung cancer, NSCLC）是世界范围内癌症死亡的主要原因^[1]^。研究^[2, 3]^表明，肺癌发生的风险与许多因素相关，年龄是其中之一，对其进一步研究能指导许多癌症的治疗。NSCLC过去被认为是一种老年疾病，年轻患者只占很小的比例^[4, 5]^。值得注意的是，年轻人中NSCLC的发病率有逐年升高的趋势^[6]^。

年轻时就发生NSCLC的患者有未知的危险因素，相关的研究也较少^[7, 8]^。与老年NSCLC患者相比，年轻患者的相对预后仍存在争议。来自美国国家癌症数据库的研究^[5]^表明，年轻NSCLC患者的总体生存率高于老年患者，发现时分期较早的患者有更大的临床获益。回顾性分析^[4]^发现，年轻的NSCLC患者的预后较差，尤其是当患者未进行驱动基因突变筛查，且应用最常用的铂类为基础的双联疗法为治疗方案时。因此，必须明确年轻NSCLC患者的分子特征和临床行为，并开发一种个体化的治疗方法来改善这一人群的预后。

以往很少有报道关注包括肺腺癌在内的年轻NSCLC患者靶向基因突变的发生率，而现在它吸引了越来越多的研究。最近的数据^[9-13]^表明，年轻的肺腺癌患者具有独特的分子特征，并具有高频率的驱动基因突变，如间变淋巴瘤激酶（anaplastic lymphoma kinase, *ALK*）基因重排。然而，在一项小规模研究^[14]^中，中国年轻人和老年人肺腺癌的癌基因突变没有发现差异。就年轻肺腺癌患者中表皮生长因子受体（epidermal growth factor receptor, *EGFR*）基因突变的几率而言，最近几项研究的结果存在争议^[9, 10, 13]^。因此，年轻患者的定义、种族、年轻肺腺癌患者的相对罕见性以及所使用的基因检测方法等因素使得这项研究更趋复杂化。

尽管在肺腺癌中发现了大量驱动基因突变，但在上述研究中，通过低通量分析对年轻患者仅研究了少数驱动基因。在这项研究中，广泛的、基于杂交捕获的下一代测序（next-generation sequencing, NGS）分析被用于确定年轻肺腺癌患者的靶向基因突变，这为我们提供了该人群基因组学更全面的信息。

## 1 材料和方法

### 1.1 患者和样本

样本经苏木素-伊红染色证实为腺癌。所有福尔马林固定的石蜡包埋组织样本均由合格的病理学专家进行检查，以确认腺癌的诊断，并确保肿瘤含量 > 30%。根据第8版国际肺癌肿瘤原发灶-淋巴结-转移（tumor-node-metastasis, TNM）分期对患者病灶进行分期^[15, 16]^。这项研究经青岛大学附属医院伦理委员会批准。所有参与研究的患者均已签署知情同意书。所有实验均按照青岛大学附属医院伦理委员会和中华人民共和国国家卫生和计划生育委员会的指导方针进行。

### 1.2 基于NGS的分析

在这项研究中，184例患者的组织样本使用基于目标基因捕获技术的NGS技术对其分析。184例患者的28个基因均存在驱动基因突变。然而，这3个版本的NGS分析所涵盖的28个基因中只有16个被选择用于进一步分析（http://dx.doi.org/10.3779/j.issn.1009-3419.2020.04.05）。这项研究重点关注OncoKB知识库中1级-3级和R1级证据类别注释的驱动基因改变。

### 1.3 统计学分析

实验数据表示为均数±标准差（Mean±SD）。采用*Pearson*卡方检验、*Fisher*精确检验和Student *t*检验分析两组间的基因突变情况和患者特征。在所有的测试中，*P*值都是双尾的。以*P* < 0.05为差异有统计学意义。

## 2 结果

### 2.1 研究设计和患者特征

对2013年1月-2019年6月于青岛大学附属医院确诊的2, 882例肺腺癌患者进行了筛查。其中45岁以下患者109例，占总病例数的3.8%。其中收集了89例患者的组织标本进行NGS分析。此外，还收集了95例在此期间接受过NGS检测的腺癌病例进行比较研究。

184例肺腺癌患者NGS检测的临床特征见表 1。所有患者的平均年龄为51岁（24岁-85岁）；63.6%（117/184）的患者为女性，71.2%（131/184）的患者从不吸烟。0期疾病占1.7%（3/184），Ⅰ期疾病占30.4%（56/184），Ⅱ期疾病占8.2%（15/184），Ⅲ期疾病占15.2%（28/184），Ⅳ期疾病占44.6%（82/184）。共收集174例原发性肿瘤组织标本和10例转移组织切片进行NGS分析。

**1:** 高通量测序法检测184例肺腺癌患者的临床特征 Clinical characteristics of 184 patients with lung adenocarcinoma with next-generation sequencing assay

Parameters	Total (*n*=184)	Younger group (*n*=89)	Older group (*n*=95)	*P*
Age (yr, range)	52 (24-85)	40 (24-45)	63 (47-85)	< 0.001
Gender Male Female	67 117	24 65	43 52	0.013
Smoking history Never Former/current	131 53	75 14	55 40	< 0.001
Stage 0 Ⅰ Ⅱ Ⅲ Ⅳ	3 56 15 28 82	3 37 10 25 14	0 19 5 3 68	< 0.001
Tumor sample source Primary Metastatic	174 10	85 4	89 6	0.744

### 2.2 目标基因谱与年龄的关系

在先前的研究中，年龄截止点40岁^[10-12, 14, 17]^、45岁^[18]^、46岁^[5]^和50岁^[13, 19]^在NSCLC的研究中都曾被定义为年轻。在这项研究中，通过比较三个年龄组的目标基因突变频率来确定一个合理的年龄临界点。如图 1所示，前两组的靶向基因突变差异无显著统计学意义（*P*=0.564）。然而，前两组与第三组（45岁以上）有统计学差异（*P* < 0.01）。值得注意的是，年龄在40岁以下和41岁-45岁之间的患者的*ALK*突变频率（24.3% *vs* 9.6%, *P*=0.072）远高于年龄在45岁以上的患者（1.1%）。因此，我们将45岁的诊断年龄作为区分年轻患者和老年患者的临界点。

**1:**
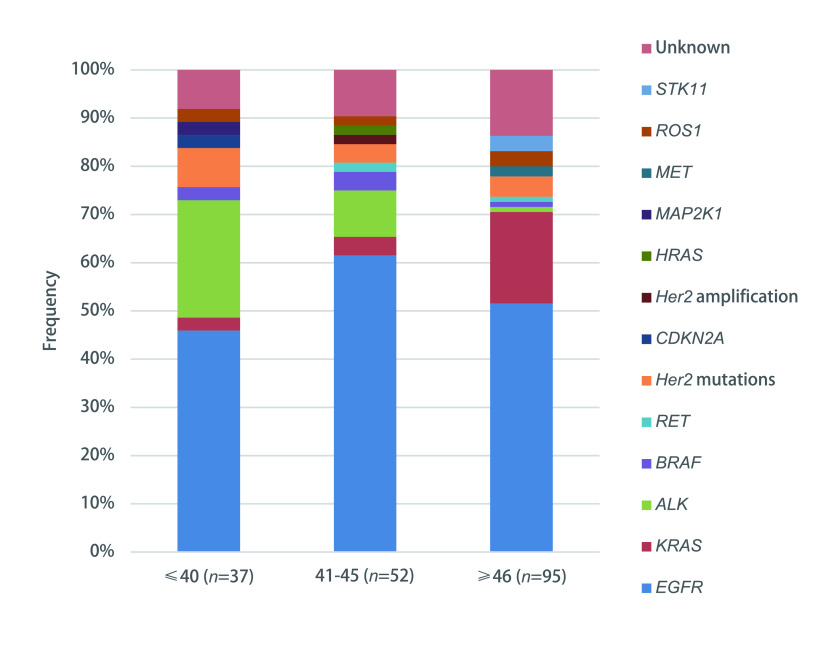
不同年龄组肺腺癌患者的基因突变。共纳入184例肺腺癌患者，用NGS法分析组织标本。比较三个年龄组的靶向基因突变频率，以确定一个年龄临界点，该临界点可以区分具有独特分子特征的年轻患者。 The genetic profiles in different age groups of patients with lung adenocarcinoma. One hundred and seventy-seven patients with lung adenocarcinoma patients were enrolled, and tissue samples were analyzed by NGS assays. The frequency of targeted genetic alterations across three age groupings was compared to determine an age cutoff point that could differentiate young patients with a distinctive molecular profile. STK11: serine/threonine kinase 11; ROS1: c-ros oncogene 1; MET: mesenchymal-epithelial transition; MAP2K1: mitogen-activated protein kinase kinase 1; HRAS: v-Ha-ras Harvey rat sarcoma viral oncogene homolog; Her2: human epidermal growth factor receptor 2; CDKN2A: cyclin dependent kinase inhibitor 2A; RET: rearranged during transfection; BRAF: v-raf murine sarcoma viral oncogene homolog B; ALK: anaplastic lymphoma kinase; KRAS: v-Ki-ras2 Kirsten rat sarcoma viral oncogene homolog; EGFR: epidermal growth factor receptor; NGS: next-generation sequencing.

### 2.3 年轻肺腺癌患者靶向基因突变的患病率

在184例肺腺癌患者中，89例年龄≤45岁的患者被分为年轻组，95例年龄≥46岁的患者被分为老年组。在年龄、性别、吸烟史、年龄分布上，年轻组与老年组有显著差异（表 1）。年轻组女性较多，吸烟者少，早期腺癌多。两组肿瘤标本来源差异无统计学意义。

91.0%的年轻患者存在驱动基因突变。在驱动基因中观察到的突变包括：*EGFR*（*n*=49, 55.1%）、*KRAS*（*n*=3, 3.4%）、*ALK*（*n*=14, 16.0%）、*BRAF*（*n*=3, 3.4%）、*ROS1*（*n*=2, 2.2%）、*RET*（*n*=1, 1.1%）、*HER2*突变（*n*=5, 5.6%）、*CDKN2A*（*n*=1, 1.1%）、*HER2*扩增（*n*=1, 1.1%）、*HRAS*（*n*=1, 1.1%）和*MAP2K1*（*n*=1, 1.1%）（图 2A）。此外，在86.3%的老年患者中发现的驱动基因突变包括：*EGFR*（*n*=49, 51.6%）、*KRAS*（*n*=18, 18.9%）、*ALK*（*n*=1, 1.1%）、*BRAF*（*n*=1, 1.1%）、*MET*（*n*=2, 2.1%）、*RET*（*n*=1, 1.1%）、*ROS1*（*n*=3, 3.2%）、*HER2*扩增（*n*=4, 4.2%）和*STK11*（*n*=3, 3.2%）（图 2B）。年轻组和老年组在NGS检测的靶向基因图谱上存在显著差异（*P* < 0.001；图 2C）。更重要的是，80.9%（72/89）的年轻患者对美国国立综合癌症网络（National Comprehensive Cancer Network, NCCN）指南（2019.V3版更新，NSCLC）批准的治疗方法存在靶向基因突变，而老年患者的这一比例为55.8%（53/95）。

**2:**
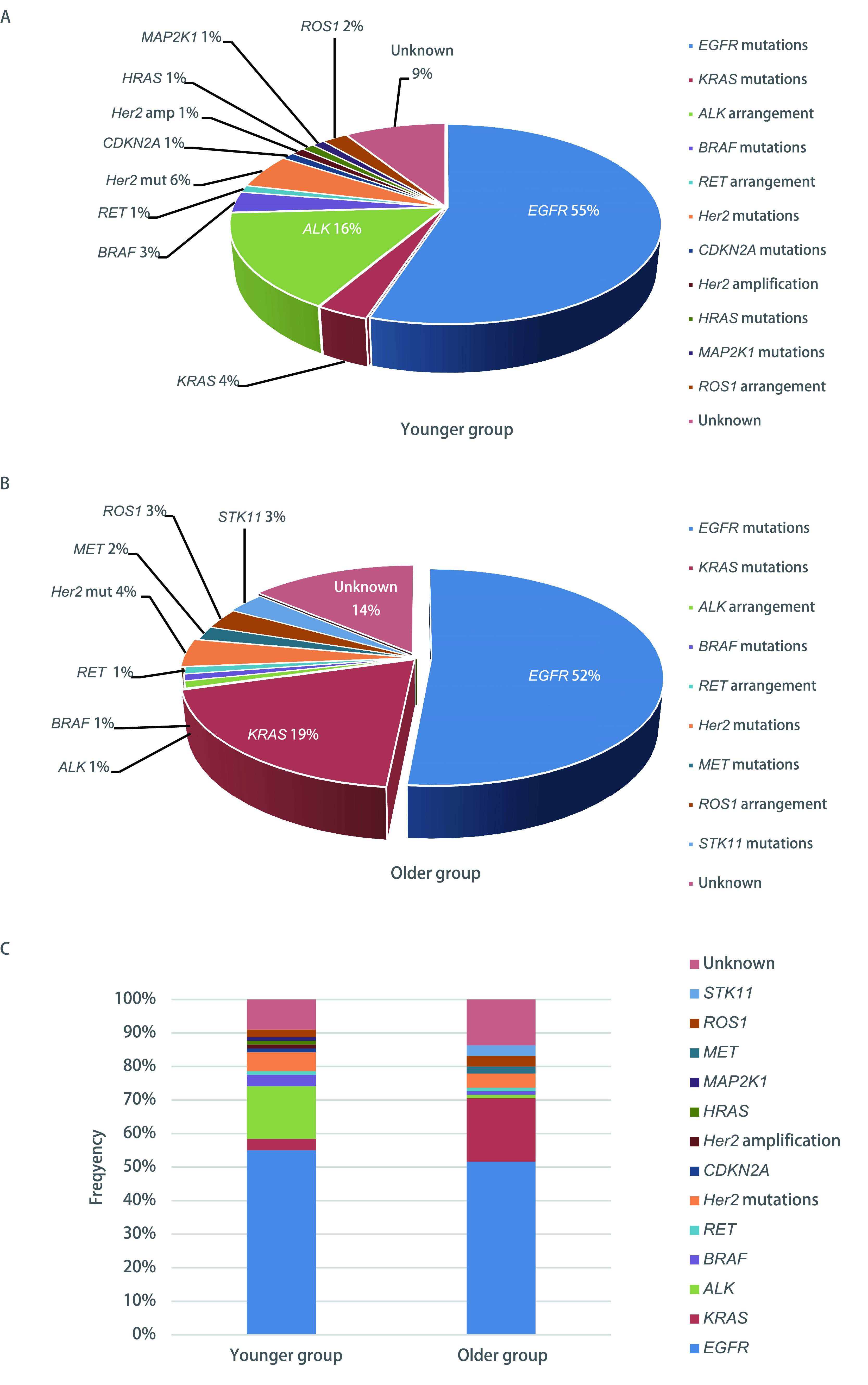
年轻和老年肺腺癌患者的靶向基因图谱。A：年龄小于45岁的年轻患者的靶向基因图谱；B：45岁以上老年患者的靶向基因图谱；C：青年组与老年组靶向基因突变频率的比较分析。 The targeted genetic profiles of younger and older patients with lung adenocarcinoma. A: The targeted genetic profile of the younger patients, aged less than 45 years; B: The targeted genetic profile of the older patients, aged more than 45 years; C: The comparative analysis of the frequency of targeted genetic alterations between the younger and the older group. amp: amplification; mut: mutation.

就所有已分析的驱动基因突变而言，只有*KRAS*突变和*ALK*重排的分布在年轻组和老年组之间存在显著差异。与老年组相比，年轻组的*KRAS*突变频率较低（3.4% *vs* 18.9%, *P*=0.002），而年轻组的*ALK*排列明显较高（15.9% *vs* 1.1%, *P* < 0.001）。年轻组和老年组之间的*EGFR*突变率相当（分别为57.6%和51.6%；*P*=0.657），而两组之间的*EGFR*突变亚型分布有显著差异（*P*=0.023）。在*EGFR*突变型肺腺癌患者中，*EGFR* 19外显子缺失及21外显子L858R突变的检出率最高，在所有突变亚型中，20外显子在年轻组中突变的频率较低（*P*=0.005），而其余突变亚型在两组之间的频率相似。

对于某些患者而言，若同时存在多个驱动基因突变，则已上市的靶向药物和实验药物均可应用。根据临床治疗效果划分，可以分为首要干预靶点及次要干预靶点，当只考虑首要靶点基因突变频率时，年轻组和老年组之间没有显著差异。然而，当分析所有单一和多基因突变时，在年轻组中可以看到更高频率的*HER2*靶向突变和低频率的*STK11*突变（*HER2* 13.8% *vs* 4.4%, *P*=0.037; *STK11* 1.1% *vs* 8.9%, *P*=0.035）。

### 2.4 对年轻肺腺癌患者*TP53*基因突变的研究

在无相应靶向药物的基因突变中，最常见的突变基因是*TP53*。*TP53*突变在年轻组和老年组中有相似的发生率（55.1% *vs* 47.3%, *P*=0.365）。然而，在*EGFR*突变的患者中，伴有*TP53*同时突变比*ALK*重排的患者更为常见（63.3% *vs* 13.2%, *P* < 0.001）。值得注意的是，在*EGFR*突变患者中，与老年患者相比，年轻患者中同时出现*TP53*突变的频率更高（64.5% *vs* 35.5%, *P* < 0.001）。见图 3。

**3:**
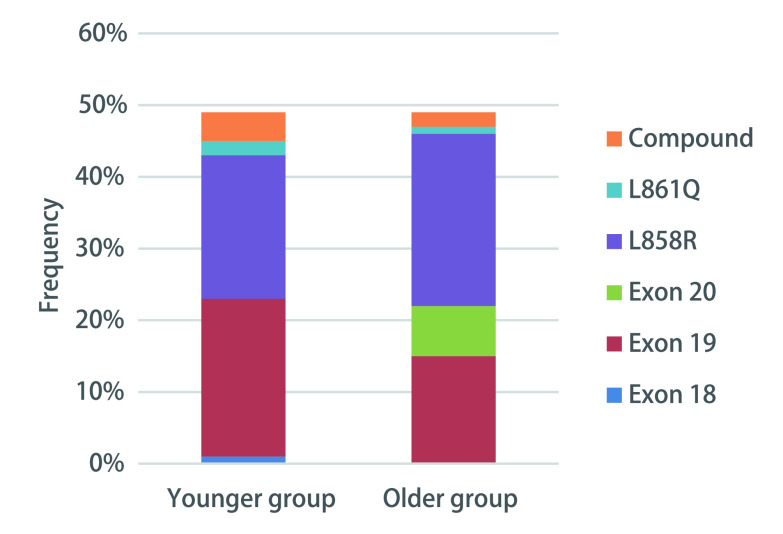
年轻和老年肺腺癌患者*EGFR*突变亚型分析 *EGFR* mutation subtypes profiles of younger and older patients with lung adenocarcinoma. Exon: expressed region.

## 3 讨论

年轻NSCLC患者已引起了越来越多的关注，其发病例占所有患者的比例不到5.0%^[5, 17, 18, 20]^。许多研究^[5, 7, 14, 16]^表明，年轻人的NSCLC具有独特的特征，女性患者、腺癌患者和从不吸烟者的比例更高，这一特点并不局限于中国，欧美也有相关报道^[15]^年轻的肺腺癌患者，尤其是晚期患者，尽管接受了更积极的治疗，但总体生存率并没有明显高于老年患者^[14]^。尽管随着基因组检测技术的进步和靶向药物的开发，肺腺癌的治疗正在迅速改变，但年轻患者的分子病理特征尚未得到很好的研究。最近的一份报告^[13]^表明，年龄越小，NSCLC（包括肺腺癌）中携带目标基因型的可能性越高。以前关于年轻肺腺癌患者分子特征的大多数数据都是不一致的，只有少数的驱动基因是通过非NGS分析进行研究的^[9, 10, 12-14]^。使用NGS进行全面的基因组分析可以发现非NGS分析漏掉的驱动基因突变，并加速针对罕见突变基因的靶向药物的开发^[21, 22]^。这项研究通过广泛靶向的NGS检测，对青年肺腺癌患者的基因特征进行了全面的鉴定。45岁以下的患者具有独特的基因突变特征，45岁可能是确定年轻人肺腺癌的有效年龄界限。值得注意的是，与老年患者相比，年轻患者存在针对NCCN批准上市的药物对应的靶向基因突变的比率要高得多。

特别的是，在年轻的肺腺癌患者中，*ALK*的突变频率较高，*KRAS*的频率较低，这与之前的研究一致^[9, 10, 13]^。然而，也有研究发现这些基因突变的发生率相似，这可能归因于年轻病例的数量少^[14, 23]^此外，这项研究表明，年龄与标准基因分型通常忽略的罕见基因突变之间存在关联。在这项研究中，年轻患者中发现了更高频率的*HER2*突变和较低频率的*STK11*突变。曾有报道称年轻腺癌患者的*HER2*基因突变频率明显高于老年组^[10]^。然而，在最近的一项研究中，*HER2*突变发生的年龄向年轻化的趋势并不显著，在这项研究中，只研究了*HER2* 20外显子中的帧插入^[13]^。上述结果强调，所有类型的*HER2*突变，包括扩增，可能是青年肺腺癌患者的一个有价值的靶点。

与*ALK*重排相比，年轻肺腺癌患者*EGFR*突变的患病率存在很大争议。与老年人相比，年轻肺腺癌患者*EGFR*突变的频率或更高或更低^[10, 13, 19]^。在这项研究中，年轻人和老年人之间没有发现*EGFR*突变类型或*EGFR*敏感突变的差异。我们的发现与另外三项针对年轻肺腺癌患者的研究^[9, 14, 22]^一致。有趣的是，在我们的研究中，能预测对临床可用的EGFR-TKIs存在抵抗的*EGFR* 20外显子突变在年轻患者中较少发生。上述关于年轻肺癌患者*EGFR*基因突变特点的研究中，相互矛盾的数据可能归因于种族、年龄界限和病理学，这需要进一步的研究。

年龄越小，携带靶向基因型的可能性越高，但与其他年龄组相比，年轻的NSCLC患者的生存率却出乎意料地低，这表明其生物学行为更具侵略性^[13]^。先前的研究也暗示年轻肺腺癌患者EGFR-TKIs的疗效较差^[19]^。年轻的*EGFR*突变腺癌患者在EGFR-TKI治疗下预后不佳的原因尚不清楚，部分原因是该亚群中有更多的脑转移^[24]^。许多机制被发现与晚期NSCLC对EGFR-TKIs的原发性耐药有关，并激活了*EGFR*突变^[25]^。除了这些机制外，已经证明，在*EGFR*突变的NSCLC患者中，通常并发的*TP53*突变降低了对EGFR-TKIs的敏感性，并使预后更差^[26-28]^。在这项研究中，年轻患者与老年患者相比，没有发现更高的*TP53*突变率，这与之前的一项发现不一致^[14]^。然而，我们发现年轻患者发生*EGFR/TP53*共突变比老年患者要高得多。更重要的是，在Canale等^[27]^的研究中部分*EGFR/TP53*共突变的患者的随访数据显示，EGFR-TKI治疗的疗效不佳，即有*TP53*突变的患者，其疾病进展的风险是*TP53*野生型患者的3倍。考虑到*EGFR/TP53*共突变的发生占年轻患者*EGFR*突变的80%以上，我们的研究可能揭示了EGFR-TKIs在这一特定人群中的预后不良和用药低效的部分原因。据报道，年轻肺腺癌患者预后较差，年轻人肺腺癌血管生成与p53表达的相关性比老年人高^[29]^。因此，埃罗替尼联合贝伐珠单抗治疗年轻人并发*EGFR/TP53*变异性肺腺癌可能是一种最佳方案，值得进一步开展深入的研究来验证这一推论^[30, 31]^。

虽然这项研究针对年轻肺腺癌患者的基因特征进行了大量NGS研究，但这项研究仍有一定局限性。研究中的患者均为中国人，来自同一机构，随访数据仅适用于少数患者。此外，46岁-50岁之间的研究对象（仅5例）的数量太少，无法作为年龄组进行分析。因此，对于具有独特遗传特征的年轻NSCLC患者，其年龄临界点的确定尚需进一步研究。

## 4 结论

年轻肺腺癌患者是一个较少被研究的人群，他们有高频率的*ALK*重排、*HER2*突变和*EGFR/TP53*共突变率。在年轻肺腺癌患者中发现的独特驱动基因需要我们更多的关注其个体化治疗。
